# Cost‐effectiveness analysis of ovarian tissue cryopreservation and transplantation for preservation of fertility in post‐pubertal oncological women submitted to high‐risk gonadotoxic chemotherapy

**DOI:** 10.1002/ijgo.14104

**Published:** 2022-02-17

**Authors:** Diego Raimondo, Ilaria Giaquinto, Manuela Maletta, Rossella Vicenti, Raffaella Iodice, Alessandro Arena, Simona Del Forno, Antonio Raffone, Jacopo Lenzi, Paolo Casadio, Renato Seracchioli

**Affiliations:** ^1^ Division of Gynaecology and Human Reproduction Physiopathology, Department of Medical and Surgical Sciences IRCCS Azienda Ospedaliero‐Universitaria di Bologna, S. Orsola Hospital, University of Bologna Bologna Italy; ^2^ Gynecology and Obstetrics Unit, Department of Neuroscience, Reproductive Sciences and Dentistry School of Medicine, University of Naples Federico II Naples Italy; ^3^ Department of Biomedical and Neuromotor Sciences University of Bologna Bologna Italy

**Keywords:** cancer, oncology, ovar*, preservation, sparing, tumor

## Abstract

**Objective:**

To study the economic impact of ovarian tissue cryopreservation and transplantation (OTC) in post‐pubertal patients who underwent high‐risk gonadotoxic chemotherapy.

**Methods:**

A decision tree model was used to determine the live birth rate and cost‐effectiveness of OTC versus non‐OTC. The incremental cost‐effectiveness ratio (ICER) was calculated. A sensitivity analysis was performed under the assumption that the costs of ovarian cortex retrieval, cryopreservation, and storage for patients with cancer might be covered by the national health system or health insurance.

**Results:**

Patients had the greatest probability of achieving live birth after high‐risk chemotherapy when they underwent OTC versus non‐OTC. Although cryopreservation of ovarian tissue results in higher live birth rates, it is always more expensive. Cost‐effectiveness increases when the majority of patients completes the path of tissue cryopreservation plus transplantation after 5 years.

**Conclusion:**

Although OCT has been demonstrated as a procedure for effective fertility preservation in fertility‐age women with cancer, no cost‐effectiveness analysis has been performed until now. This model could help healthcare systems to allocate coverage for OCT.

## 1 INTRODUCTION

Premature ovarian insufficiency (POI) is a clinical syndrome defined by loss of ovarian activity before the age of 40 years. POI is characterized by amenorrhea or oligomenorrhea with raised gonadotropins, low estradiol, and severe consequences on fertility.^1^

The prevalence of POI in the general population is approximately 1%. In recent years, the incidence of iatrogenic POI in women with cancer has been growing: 4% of fertile patients receive diagnoses of malignancy and potentially sterilizing gonadotoxic treatments, including chemotherapy.^1^, ^2^

Chemotherapy can be categorized as high (HRC) or low risk (LRC) depending on its gonadotoxicity.^2^ After a diagnosis of cancer, both LRC and HRC decrease the probability of a live birth. The maximum probability of a live birth at a horizon age of 5 years are 55% for LRC and under 20% for HRC at any age.^3^

Preserving the ability to have biological children is the most important goal for many survivors of cancer.^4^ The American Society of Clinical Oncology (ASCO) states that healthcare providers caring for oncologic patients should address the possibility of infertility as early as possible before treatment starts.^5^ Before gonadotoxic cancer treatments, post‐pubertal patients with the desire to have future children can opt for two main different methods of preservation of fertility to ensure their own homologous fertility: oocyte cryopreservation after ovarian stimulation and ovarian tissue cryopreservation (OTC).^3^, ^6^

Oocyte cryopreservation has been shown to be a reproducible, safe, and effective technique for several years. However, it has two important limitations: first, oocyte cryopreservation can delay the start of cancer treatment; and second, in some cancers ovarian stimulation should be avoided.^7^

In these cases, OTC is considered as the unique option for preservation of fertility, with an increasing number of successful and safe reports.^4^, ^7^, ^8^, ^9^, ^10^, ^11^, ^12^, ^13^ Moreover, unlike oocyte cryopreservation, OTC also allows restoration of ovarian hormonal function as long as the graft is active.^14^

Cost‐effectiveness analysis (CEA) can help healthcare systems, practitioners, and patients to weigh the benefits and costs of preservation of fertility, and to highlight critical points of the clinical protocols in health resource‐limited settings.^15^ In 2017, Lyttle Schumacher et al.^3^ performed a CEA of oocyte cryopreservation before chemotherapy, reporting higher costs and live birth rates (LBRs) in women adopting oocyte cryopreservation compared to patients of all ages not adopting oocyte cryopreservation; in particular, oocyte cryopreservation was most cost‐effective for women undergoing HRC at younger ages. On the contrary, it is believed that no CEA study for an OTC program is present in the literature to date.

The aim of the present study was to create a decision tree model to determine the LBR and cost‐effectiveness of OTC versus non‐OTC in fertile women with cancer in whom gonadotoxic chemotherapy cannot be postponed or with contraindications to ovarian stimulation.

## 2 MATERIAL AND METHODS

Costs included in the CEA were drawn from previously published fees for removal of tissue, cryopreservation and storage for 5 years, and transplantation of thawed tissue.^16^ These fees were equal to €5000, €4000, and €5000, respectively. Transportation costs (in the range of €100–€400) were not taken into account, considering their poor impact on total costs. In vitro fertilization (IVF) costs were not taken into account either, under the assumption that the probability of IVF is the same for OTC and non‐OTC patients. The analysis was conducted from the perspective of the payer.

The clinical outcome under study was the LBR in a time horizon of 5 years after treatment for cancer.

LBR values were taken from published literature. More specifically, the probability of a live birth after OTC plus transplantation was set equal to 0.33, as estimated by pooling the results from Diaz‐Garcia (*n* = 44),^4^ Meirow (*n* = 20),^17^ Poirot (*n* = 24),^18^ and Liebenthron (*n* = 30)^19^ (Table S1). More specifically, overall LBR was given as [[(0.23 × 44) + (0.50 × 20) + (0.33 × 24) + (0.37 × 30)]/118 = 0.33]. It was decided to base the present analysis on these studies because they specifically evaluated fertility results in oncological fertile women who underwent chemotherapy and orthotopic auto‐transplantation of ovarian tissue and desiring pregnancy. The weighted average age at diagnosis and treatment of the four study samples was 31 years, with a horizon age of 31 + 5 = 36 years. Because the vast majority of patients (>90%) experienced POI after chemotherapy, it was inferred that virtually all of them underwent HRC. This means that the target population of the present CEA includes patients undergoing HRC at the age of 31 years and desiring pregnancy.

**FIGURE 1:**
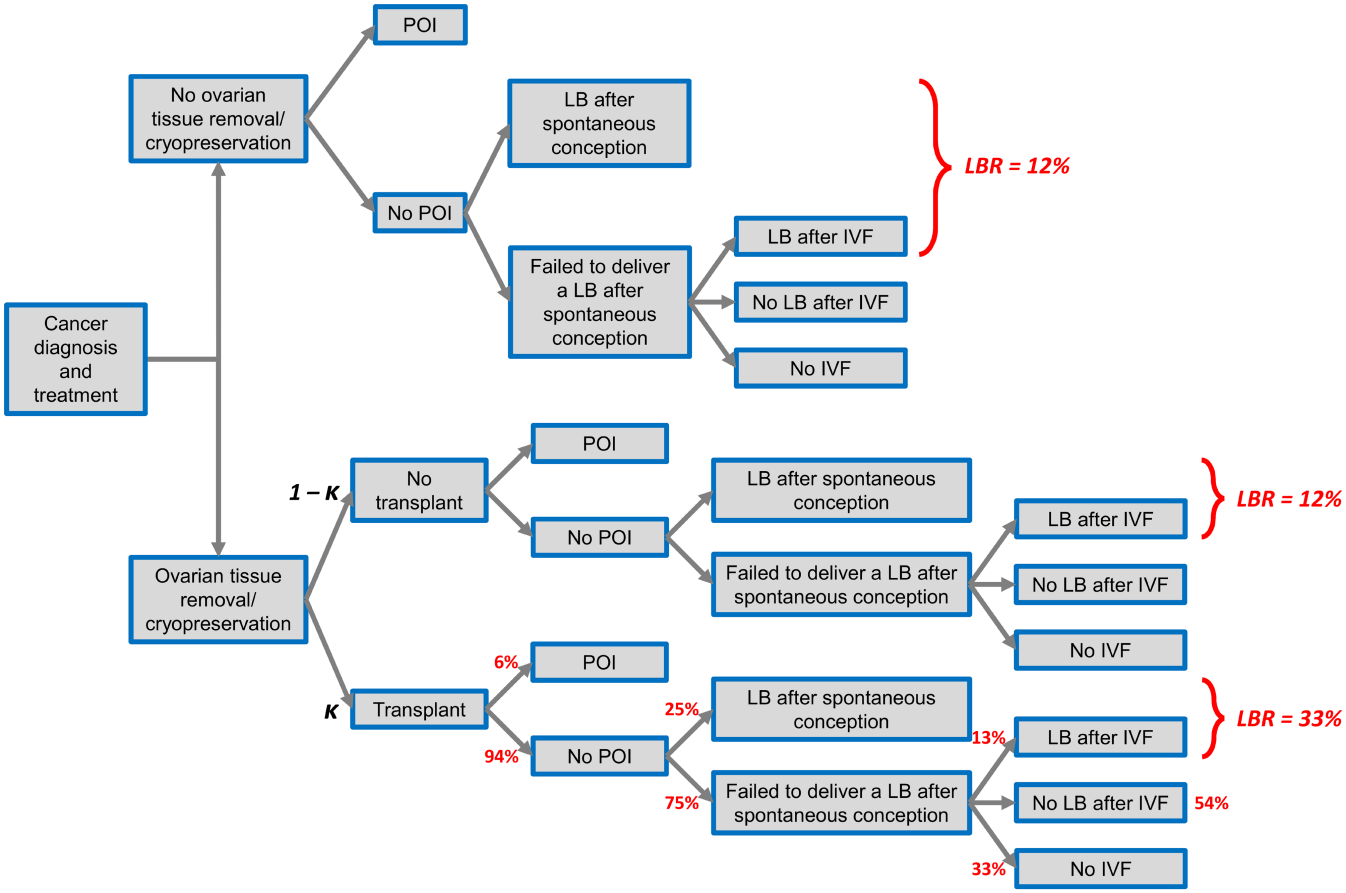
Decision tree model for cost‐effectiveness analysis of ovarian tissue removal and cryopreservation. Abbreviations: IVF, in vitro fertilization; LB, live birth; LBR, live birth rate; POI, premature ovarian insufficiency

For all these reasons, the probability of a live birth in the comparator group (no tissue removed and cryopreserved) was specified as 0.12, which is the LBR described by Lyttle Schumacher et al.^3^ for 31‐year‐old women who do not undergo any preservation of fertility after HRC. The same probability of 0.12 was assumed for patients who do have their tissue removed (ovarian biopsy) and cryopreserved, but do not undergo reimplantation in a time horizon of 5 years after cancer treatment (Figure 1).

Figure 1 provides a visual representation of the decision tree that was used to structure the CEA. The resulting incremental cost‐effectiveness ratio (ICER) was the ratio of overall difference in cost between choosing tissue removal and cryopreservation versus not choosing tissue removal and cryopreservation, divided by the incremental difference in LBR:

$$\mathrm{ICER}=\frac{{\mathrm{cost}}_{1}-{\mathrm{cost}}_{0}}{{\mathrm{LBR}}_{1}-{\mathrm{LBR}}_{0}}$$

where (1) is the experimental group and (0) is the comparator group. *ICER* expresses the cost per additional live birth when undergoing ovarian tissue removal and cryopreservation. No discount rate was applied to costs and LBRs.

To understand to what extent the difference in the distribution of clinical outcomes and costs in the tree affects the results of the CEA, a scenario analysis was performed by varying the proportion of individuals that end up following the path of cryopreservation plus transplantation from a minimum of 0.05 to a maximum of 0.95. Moreover, because the LBR of 0.33 had been calculated from a relatively small‐pooled sample of 118 women undergoing tissue transplantation,^4^ the CEA on the 95% lower and upper confidence bounds of 0.33 was replicated to account for uncertainty in this probability estimate. In other words, the analysis was repeated by assuming higher and lower LBR values distant from 0.33, but still compatible with the data drawn from the literature. These two bounds were equal to 0.24 and 0.45, and were obtained using the 95% confidence interval for a Poisson mean. Lastly, to generalize the results of the CEA, a sensitivity analysis was performed under the assumption that the costs of ovarian cortex retrieval, cryopreservation, and storage for patients with cancer might be free, that is, covered by the national health system or health insurance. Therefore, the only cost for the CEA was for the reimplantation procedure when patients come back to use the cryopreserved ovarian tissue (€5000). This economic evaluation was based on a literature review, and theoretical statistical modeling was applied on a hypothetical cohort of patients. It did not require approval by the Institutional Research Ethics Board. All analyses were carried out using Microsoft Excel (2016) and Stata software version 15 (StataCorp LLC., College Station, TX, USA).

## 3 RESULTS

As shown in Figure 1, 94% of transplanted patients after HRC resume menstruation or improve their menopausal symptoms. Of them, 25% conceive naturally and deliver a live birth, while 75% fail to have a live birth: 67% of them go on to IVF, with only 13% succeeding with it, while 33% do not go on to IVF.

Patients have the greatest probability of achieving a live birth after HRC when they undergo ovarian cryopreservation and transplantation (LBR = 0.33) versus non‐cryopreservation (LBR = 0.12) (Figure 1). Assuming a utilization rate of 5%, the overall LBR in the experimental cohort of patients undergoing OTC is (0.95 × 0.12) + (0.05 × 0.33) = 0.13, with an estimated cost of 0.95 × (€5000 + €4000) + 0.05 × (€5000 + €4000 + €5000) = €9250. The resulting ICER is €887 254 per additional live birth when undergoing ovarian tissue removal and cryopreservation.

**FIGURE 2:**
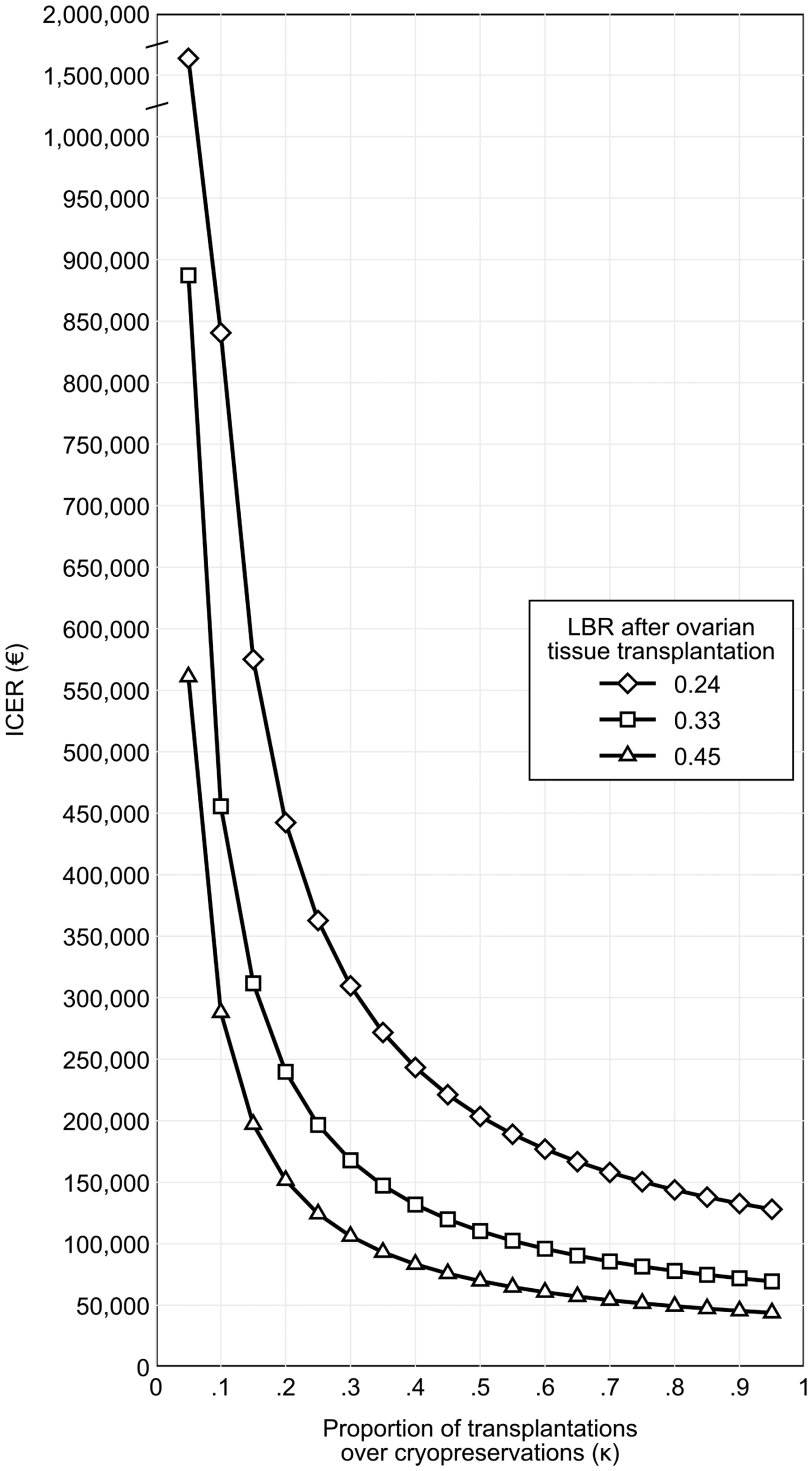
ICER according to the proportion of patients that have their ovarian tissue reimplanted after 5 years of cryopreservation (*x*‐axis) and to the probability of live birth after ovarian tissue transplantation. The lower and upper bounds of the LBR are the 95% confidence limits of the LBR of 0.33 estimated by pooling the results from Diaz‐Garcia,^4^ Meirow,^17^ Poirot,^18^ and Liebenthron.^19^ Abbreviations: ICER, incremental cost‐effectiveness ratio; LBR, live birth rate

As shown in Figure 2, the cost‐effectiveness increases when the majority of patients completes the path of tissue cryopreservation plus transplantation after 5 years. When the percentage of patients completing the path is about 20%, the ICER starts to decrease substantially (€239 798). At 60% of patients completing the path, the ICER is €95 919. All the ICER values illustrated in Figure 2 are listed in Table S2.

When only the cost of transplantation is retained in the analysis, the ICER of patients who undergo tissue cryopreservation before HRC is €23 980, ranging from €15 160 (assuming a LBR = 0.45) to €44 238 (assuming a LBR = 0.24).

## 4 DISCUSSION

Advances in oncological treatments have significantly improved cure rates of many young cancer patients, but the quality of life of these women will not be satisfying until the question of their fertility after gonadotoxic treatment will be systematically taken into account.^20^

In order to make procedures to preserve fertility as an integral part of cancer management, it is crucial to know their economic health costs as well.

It is believed that this is the first study assessing the cost‐effectiveness of OTC, analyzing effectiveness in terms of LBR and costs before gonadotoxic treatment in adult women with cancer desiring pregnancy and comparing them to non–fertility‐preserving procedures. Attention was focused on fertile female candidates for HRC, since it carries a considerable chance of POI and infertility (with estimated live births <20%) regardless of their age.^3^

The present study showed that OTC is more effective than non‐OTC path in terms of LBR (0.33 vs. 0.12); however, OTC in the present model is very expensive up to ICER at nearly €900 000.

An important point in our analysis is the limited number of patients who come back to use the cryopreserved material,^4^, ^21^ leading to a negative impact in the ICER of the procedure. Several causes in the period between ovarian retrieval and transplantation can justify this poor rate, including the following: (1) loss of desire for pregnancy; (2) cancer relapse; (3) death of the patient; (4) financial reasons; (5) fear of cancer recurrence due to contamination of ovarian retrieval; (6) onset of spontaneous pregnancies; and (7) partner status, and if no current partner, the further barrier of accessing donor sperm.

The better way to maximize the cost‐effectiveness is to increase the number of patients following the path of tissue cryopreservation plus transplantation after 5 years. For example, at 20% and at 60% of patients completing the path (vs. 5.5%) there is a reduction in cost per live birth (ICER) down to €239 798 and €95 919, respectively.

To improve the ICER, it is necessary to increase the rate of completed OTC programs through a detailed patient selection and counseling before starting the protocol, careful follow‐up, and continuous psychological support.^21^

From experiences of fertility centers, it is necessary to include only patients with strict restrictions based on age or ovarian reserve, preferably before the first cycle of chemotherapy and without other factors affecting fertility.^22^

In our sensitivity analysis regarding healthcare coverage, in which the costs of retrieval, cryopreservation, and 5‐year storage were removed, the ICER for patients who undergo OTC before HRC in our model was €23 980. Despite this scenario resulting in a decrease in cost per live birth of €863 274, the cost remains too high compared to Italy's per capita gross domestic product (€29 639, The World Bank 2019.^23^ Women's projects and family goals surely influence the measure of willingness to pay, but this estimation is impossible due to lack of data.^15^

Comparing OTC with oocyte cryopreservation in oncological patients submitted to HRC at the same age of diagnosis as shown by Lyttle Schumacher et al.,^3^ oocyte cryopreservation has a higher LBR (0.61) and is more cost‐effective than OTC (with an ICER of approximately €35 000). As highlighted in our reference study,^4^ OTC showed a lower LBR compared to oocyte cryopreservation for the following reasons: (1) broader selection at the beginning of the OTC program including older patients aged over 35 years; (2) limited follow‐up; and (3) possible dysfunctional folliculogenesis and asynchrony between the oocyte and the granulosa cells altering oocyte morphology. However, despite its disadvantageous ICER compared to oocyte cryopreservation, OTC remains the only option for the preservation of fertility in women in whom chemotherapy cannot be postponed or with contraindications to ovarian stimulation.^7^ Furthermore, OTC in the majority of patients allows resumption of ovarian function for 3–7 years, which can eventually be postponed through two or more tissue transplantations.^8^

Although the present study is a useful starting point to manage health resources and counsel patients to consider OTC for the preservation of fertility in oncological patients, it has some limitations. First, taking the post‐transplantation LBR from a pooled sample of 118 women makes the present ICER estimates uncertain. However, despite OTC has recently been considered a standard method of FP that is particularly relevant for women in need of urgent therapy, the procedure is carried out only in few centers leading to low‐volume studies including both oncological and non‐oncological indications.^4^, ^7^, ^8^, ^9^, ^10^, ^11^, ^12^, ^13^, ^17^, ^18^, ^19^, ^24^ Nevertheless, the paucity of economic data about OTC programs needs clinical and economic investigations to improve its knowledge and clinical use.

Second, deriving clinical data from different independent sources is a drawback that undermines the internal and external validity of the results of the present study. As already stated, larger clinical studies in different healthcare settings should be undertaken to evaluate the clinical effectiveness and economic feasibility of ovarian tissue cryopreservation.

Third, the economic benefits of OTC regarding hormonal ovarian activity were not evaluated in the present CEA. After orthotopic or heterotopic reimplantation of ovarian tissue, hormonal ovarian activity is restored or ameliorated in more than 95% of cases.^7^ The lack of relative data on bone, cardiovascular, neurological, sexual and genitourinary status, and quality of life after transplantation does not allow for the evaluation of specific direct and indirect health costs of POI.

Fourth, likewise, due to missing data on available literature regarding psychological and functional (work and social life) consequences of infertility in women with cancer after gonadotoxic therapies with and without OTC, it was also not possible to evaluate these aspects.

Fifth, prepubertal girls who could benefit from OTC were not considered in the present study, since they were not included in the reference studies.^4^ Other studies are needed to evaluate the ICER in this particular group of patients.

Finally, the model in the present study did not evaluate specifically the impact of radiation exposure on fertility due to lack of robust data regarding the LBR in patients with cancer submitted to radiotherapy with or without OTC.

In conclusion, despite these drawbacks, this model could help practitioners facilitate counseling about costs and outcomes associated with OTC and help healthcare systems allocate coverage for this program, shedding light on its critical points.

## AUTHOR CONTRIBUTIONS

DR: study conception, study design, study methods, data extraction, data analysis, manuscript preparation; GI: study conception, study design, study methods, data extraction, data analysis, manuscript preparation; MM: study conception, study design, study methods, data extraction, data analysis, manuscript preparation; VR: study design, study methods, data analysis, manuscript preparation; IR: study conception, study design, study methods, data analysis, manuscript preparation; AA: data analysis, manuscript preparation; DFS: study design, data analysis, manuscript revision, methods supervision; AR: study conception, study design, manuscript revision, methods supervision, whole study supervision; LJ: study conception, study design, manuscript revision, methods supervision, whole study supervision; PC: data analysis, manuscript revision, validation; SR: study conception, study design, manuscript revision, methods supervision, whole study supervision. All authors approved the final version to be published, and agreed to be accountable for all aspects of the work in ensuring that questions related to the accuracy or integrity of any part of the work are appropriately investigated and resolved.

## CONFLICTS OF INTEREST

The authors have conflicts of interest.

## Supporting information


Table S1

Table S2

## Data Availability

Data available on request from the authors.
