# Supplementary material for: Cost‐effectiveness analysis of ovarian tissue cryopreservation and transplantation for preservation of fertility in post‐pubertal oncological women submitted to high‐risk gonadotoxic chemotherapy
Source: Int J Gynaecol Obstet. 2022 Feb 17;159(1):116–21. doi: 10.1002/ijgo.14104 (PMC9540266; doi:10.1002/ijgo.14104)
Supplement: Supplementary file 1 — Table S1 [file IJGO-159-116-s002.docx]

**Supplementary Table 1.** Details of the reference studies.^a^

| **Item** | **Meirow (2016)** | **Diaz (2018)** | **Liebenthron (2019)** | **Poirot (2019)** | **Total** |
| --- | --- | --- | --- | --- | --- |
| No. of patients | 20 | 44 | 30 ^b^ | 24 | 118 |
| Age at retrieval (years) | 29±8 | 34±7 | 31±5 | 27±6 | 31 |
| Active tissue after transplantation | 19/20 (95) | 43/44 (98) | 28/30 (93) | 21/24 (88) | 111/118 (94) |
| LBR | 10/20 (50) | 10/44 (23) | 11/30 (37) | 8/24 (33) | 39/118 (33) |
| LBR after spontaneous conception | 4/10 (40) | 5/10 (50) | 11/11 (100) | 8/8 (100) | 28/39 (72) |
| LBR after IVF | 6/10 (60) | 5/10 (50) | 0/11 (0) | 0/8 (0) | 11/39 (28) |

Abbreviations: IVF, in vitro fertilization; LBR, live birth rate.

^a^ Values are given as number (percentage) or mean ± SD.

^b^ Patients who underwent orthotopic transplantation after premature ovarian insufficiency and without radiotherapy of the pelvis.
