# Supplementary material for: Cost‐effectiveness analysis of ovarian tissue cryopreservation and transplantation for preservation of fertility in post‐pubertal oncological women submitted to high‐risk gonadotoxic chemotherapy
Source: Int J Gynaecol Obstet. 2022 Feb 17;159(1):116–21. doi: 10.1002/ijgo.14104 (PMC9540266; doi:10.1002/ijgo.14104)
Supplement: Supplementary file 2 — Table S2 [file IJGO-159-116-s001.docx]

**Supplementary Table 2.** ICER according to the proportion *κ* of patients that have their ovarian tissue reimplanted after 5 years of cryopreservation (*x*-axis) and to the probability of live birth after ovarian tissue transplantation.^a^

| *κ* | LBR after transplantation | | |
| --- | --- | --- | --- |
|  | 0.45 | 0.33 | 0.24 |
| 0.05 | 560 919 | 887 254 | 1 636 823 |
| 0.10 | 288 039 | 455 617 | 840 531 |
| 0.15 | 197 080 | 311 738 | 575 100 |
| 0.20 | 151 600 | 239 798 | 442 385 |
| 0.25 | 124 312 | 196 635 | 362 755 |
| 0.30 | 106 120 | 167 859 | 309 669 |
| 0.35 | 93 126 | 147 305 | 271 751 |
| 0.40 | 83 380 | 131 889 | 243 312 |
| 0.45 | 75 800 | 119 899 | 221 192 |
| 0.50 | 69 736 | 110 307 | 203 497 |
| 0.55 | 64 774 | 102 459 | 189 019 |
| 0.60 | 60 640 | 95 919 | 176 954 |
| 0.65 | 57 141 | 90 386 | 166 745 |
| 0.70 | 54 143 | 85 642 | 157 994 |
| 0.75 | 51 544 | 81 531 | 150 411 |
| 0.80 | 49 270 | 77 934 | 143 775 |
| 0.85 | 47 263 | 74 761 | 137 920 |
| 0.90 | 45 480 | 71 940 | 132 715 |
| 0.95 | 43 884 | 69 415 | 128 059 |

Abbreviations: ICER, incremental cost-effectiveness ratio; LBR, live birth rate.

^a^ The lower and upper bounds of the LBR are the 95% confidence limits of the LBR of 0.33 estimated by pooling the results from Diaz-Garcia [4], Meirow [17], Poirot [18], and Liebenthron [19].
